# A randomized controlled trial of acupoint application for postherpetic neuralgia

**DOI:** 10.3389/fpain.2025.1637449

**Published:** 2026-01-05

**Authors:** Shizhuang Zhu, Chengzhang Wu, Bingyu Pu, Jingke Nie, Hongyan Song, Xiuzhen Su, Jianxin Zhang, Jian Wang, Dianhui Yang

**Affiliations:** 1 School of Acupuncture and Massage, Shandong University of Traditional Chinese Medicine, Jinan, China; 2 Acupuncture and Massage Department, Stockton University, Galloway, NJ, United States; 3 Acupuncture and Massage Department, Shandong Institute of Traditional Chinese Medicine Hospital, Jinan, China; 4 Acupuncture and Massage Department, Beijing University of Traditional Chinese Medicine Zaozhuang Hospital, Zaozhuang, China; 5 Acupuncture and Massage Department, Weifang Hospital of Traditional Chinese Medicine, Weifang, China; 6 Acupuncture and Massage Department, Shandong University Second Hospital, Jinan, China; 7 Acupuncture and Massage Department, Shandong University of Traditional Chinese Medicine Hospital, Jinan, China

**Keywords:** acupressure, PHN, inflammatory factors, T-cell subpopulations, neurotransmitters, randomized controlled trial

## Abstract

**Objective:**

To assess the clinical efficacy of acupressure application in treating postherpetic neuralgia (PHN) of the qi (vital energy) stagnation and blood stasis type, as well as its impact on blood inflammatory factors, T-cell subpopulations, and neurotransmitter levels.

**Methods:**

A total of 134 patients diagnosed with PHN characterized by qi stagnation and blood stasis were randomly assigned to either the treatment group (67 patients, including 10 dropouts) or the control group (67 patients, including 7 dropouts). In addition to standard health education, the treatment group received treatment with anti-swelling and analgesic patches in combination with Chinese medicine fine powder acupoint patches. The control group, on the other hand, received placebo anti-swelling and analgesic patches along with placebo Chinese medicine fine powder acupoint patches. Both groups underwent treatment at specific acupoints including bilateral Sanyinjiao, Shenque, and Ashi points. The Sanyinjiao acupoint was stimulated for 30 min per session, once every 7 days. The Shenque and Ashi acupoints were stimulated for 6–8 h daily for a single session. Patients in both groups were assessed before and after treatment using the Visual Analog Scale (VAS) score, Traditional Chinese Medicine (TCM) syndrome score, Pittsburgh Sleep Quality Index (PSQI) score, 36-item Short Form Health Survey (SF-36) score, inflammatory factors including monocyte chemotactic protein-1 (MCP-1), interleukin 6 (IL-6), tumor necrosis factor alpha (TNF-α), T-cell subpopulations cluster of differentiation 3 (CD3+), cluster of differentiation 4 (CD4+), cluster of Differentiation d (CD8+), as well as neurotransmitters 5-hydroxytryptamine (5-HT), substance P (SP), and β-endorphin (β-EP). Changes in content were observed, and any adverse reactions were monitored. Clinical efficacy was evaluated after a 4-week treatment period.

**Results:**

After 4 weeks of treatment, the VAS score, TCM syndrome score, PSQI score, levels of MCP-1, IL-6, TNF-α, CD8+, 5-HT, and SP in both groups significantly decreased compared to pre-treatment levels (*P* < 0.05). Moreover, these parameters were lower in the treatment group than that in the control group (*P* < 0.05). Conversely, the SF-36 scores, CD3+, CD4+, and β-EP levels were significantly higher in post-treatment analyses compared to that at the baseline (*P* < 0.05). In addition, these values were higher in the treatment group than that in the control group (*P* < 0.05). The total effective rate in the treatment group was 84.21%, significantly surpassing the control group's rate of 61.67% (*P* < 0.05).

**Conclusion:**

Acupuncture point paste therapy for PHN of the qi stagnation and blood stasis type has been shown to decrease levels of MCP-1, IL-6, TNF-α, CD8+, 5-HT, and SP in the blood. Simultaneously, it increases levels of CD3+, CD4+, and β-EP. This treatment leads to amelioration of pain symptoms and TCM symptoms and enhancement of sleep quality and overall quality of life. The therapeutic outcomes are both safe and dependable.

**Clinical Trial Registration:**

CONSORT ChiCTR2200056614.

## 1 Introduction

Postherpetic neuralgia (PHN) is the predominant complication of herpes zoster (HZ) resulting from varicella-zoster virus infection. It manifests as neuropathic pain in the region of prior skin lesions following resolution of the acute herpes episode, persisting for months to years (1). The duration of pain should be at least 3 months after the onset or recovery of HZ, and it must match the nerve distribution area of the affected skin segment or cranial nerve (2).

**Figure 1:**
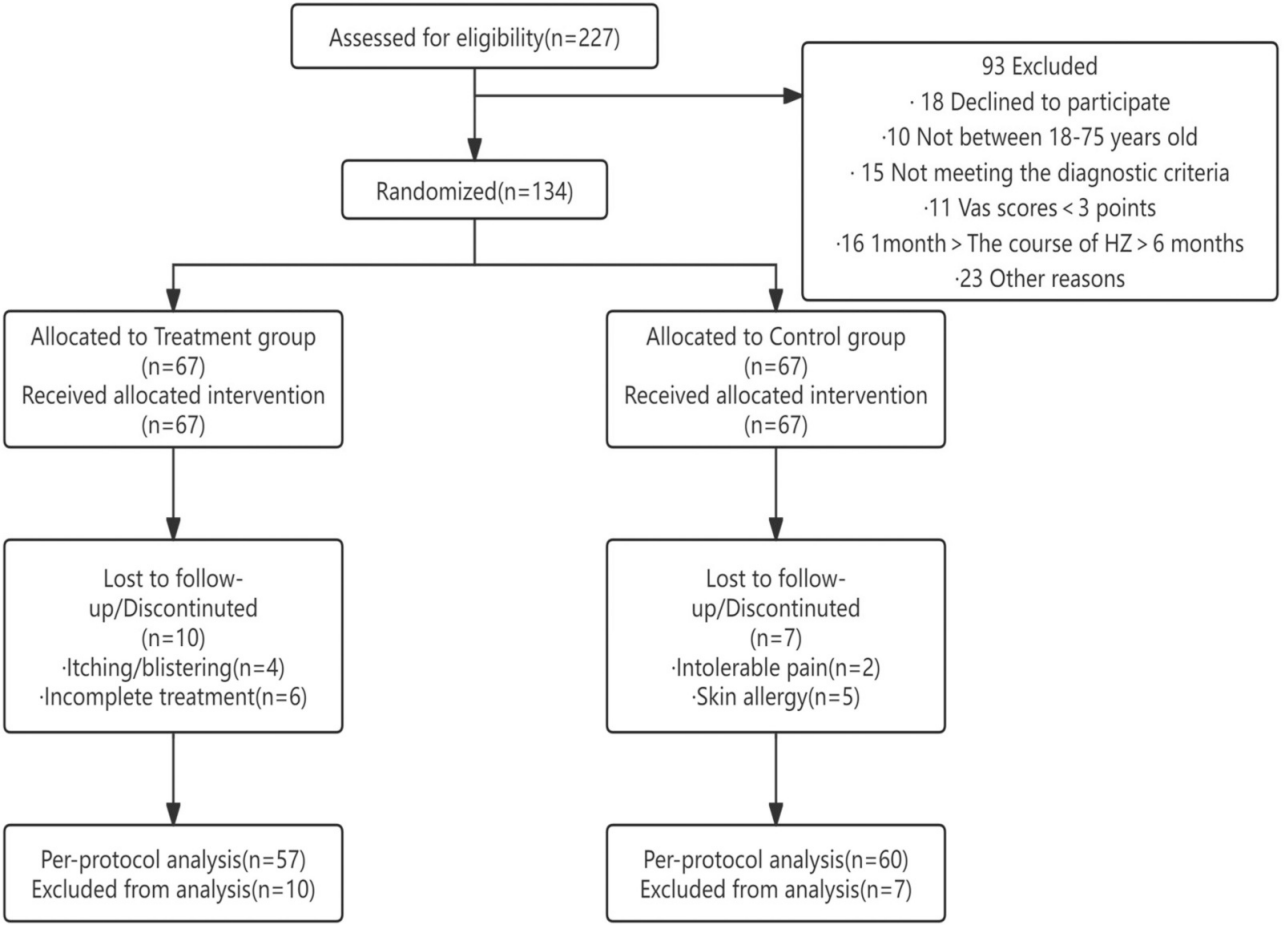
Flow in patients randomized to receive acupoint patch and placebo acupoint patch.

In Traditional Chinese Medicine (TCM), PHN is categorized as “snake string sores” and “tangled waist dan.” It is believed that blood stasis is the primary pathological factor in this condition (3). The stagnation of qi and blood, uncleared toxicity, impaired blood flow, and resulting pain are key aspects of PHN pathogenesis. Treatment aims to address these factors by promoting blood circulation, resolving blood stasis, regulating qi flow, and alleviating pain.

Contemporary medical understanding suggests that PHN is influenced by factors involving both the peripheral and central nervous systems. It is commonly attributed to viral infection of the ganglion innervating the affected pain region. Treatment typically involves the administration of tramadol, gabapentin, pregabalin, tricyclic antidepressants, or long-acting opioids (4). However, the effectiveness of these treatments is often compromised by their potential side effects (5).

TCM offers various modalities for treating diseases, including Chinese herbal medicine, acupuncture, fumigation, external applications, hot ironing, needle knife techniques, and acupoint injections (6). Acupoint application, a traditional external therapy in TCM, has shown promising results in the management of PHN (7), including pain relief, improved sleep quality, and enhanced quality of life. However, the available evidence is weakened by significant methodological limitations, as most trials were non-randomized, non-blinded, lacked placebo controls, and were restricted to single-center settings (8, 9). Therefore, this study was designed as a multicenter, randomized, double-blind, placebo-controlled trial to assess the clinical efficacy and safety of acupoint application in treating PHN of the qi stagnation and blood stasis type. The findings of this trial are presented herein.

## 2 Information and methods

This trial has been registered with the Chinese Clinical Trial Registry Center (Registration Number: ChiCTR2200056614), and it adheres to the CONSORT guidelines. All patients have signed the informed consent form. This study was approved by the Ethics Committee of the Affiliated Hospital of Shandong University of TCM [Ethics Approval No. (2022) Lun Audit No. (002)-KY].

### 2.1 General information

Patients were recruited successively from each participating center. From July 2022 to July 2023, a total of 134 patients diagnosed with PHN of the qi stagnation and blood stasis type were recruited from five outpatient departments: Shandong University of TCM Hospital, Shandong Institute of TCM Hospital, Beijing University of TCM Zaozhuang Hospital, Weifang Hospital of TCM, and Shandong University Second Hospital. The recruited patients received treatment at their respective branch centers. Among them, 30 cases were recruited at Shandong University of TCM Hospital and 26 cases were recruited at each of the other four centers. Each patient at the subcenters was randomly assigned to the treatment group or the control group (in a 1:1 ratio) using a random number table method. The patients, physicians, outcome assessors, and statisticians were blinded to treatment allocation.

### 2.2 Diagnostic criteria

Western medicine diagnostic criteria were defined according to “The IASP classification of chronic pain for ICD-11: chronic neuropathic pain (2).”

TCM diagnostic criteria were defined according to “Guidelines for the diagnosis and treatment of snake string sores in Chinese medicine (2014 revised edition) (10).”

The primary symptom is intolerable localized pain after the rash subsides, which may radiate to nearby areas.

The secondary symptoms include the following: (1) lumps, which may disperse or gather at times; (2) distension of the chest, hypochondrium, and abdomen; (3) purplish tongue or petechiae, yellow or white moss; and (4) stringy pulse. A diagnosis requires the presence of the primary symptom in combination with at least two of the secondary symptoms.

All the patients met the definition of PHN under international standards, while fulfilling the Chinese consensus criteria.

### 2.3 Inclusion criteria

Patients that met the following criteria were included in the study: (1) diagnosed with PHN per the criteria above; (2) aged between 18 and 75 years; (3) recorded a Visual Analog Scale (VAS) score ≥3; (4) indicated duration of HZ between 3 and 6 months; (5) Voluntarily participated in this trial and signed an informed consent form.

### 2.4 Exclusion criteria

Patients that met the following criteria were excluded: (1) hepatic or renal failure, (2) allergy to study medications, (3) coagulation disorders, (4) cognitive dysfunction, (5) pregnant or lactating women, (6) psychoneurologic disorders, and (7) continuous use of other analgesic drugs.

### 2.5 Criteria for withdrawal and dropout

Patients who showed (1) poor compliance during the treatment or use of medications that interfere with the results of this study; (2) pain intolerance with voluntary withdrawal; or (3) completion of the trial but use of less than 80% of the prescribed dosage were also excluded.

### 2.6 Treatment

Both groups received standardized health education regarding PHN, including on low-salt and light diet, regular work and rest, avoidance of smoking and limiting alcohol, moderate exercise, staying positive, avoiding exertion, and so on. The treatment group received acupoint application using anti-swelling and analgesic patches and Chinese medicine fine powder acupoint patches. The control group received placebo anti-swelling and analgesic patches and placebo Chinese medicine fine powder. The two groups were treated by the department of the physician with a practicing physician's license. To maintain the consistency of the operation, each patient received acupoint application from the same physician. Each physician received standardized training on the operation procedures for applying acupressure patches and passed the assessment.

#### 2.6.1 Treatment group

The acupuncture points focused on included bilateral Sanyinjiao (SP6), and Shenque (RN8) and Ashi points (the two most painful points).

For the preparation of materials, the following were used: Fine powder of asarone and white mustard seed (for Sanyinjiao points), fine powder of calamus and chuangxiong (for Shenque point), fine powder of galangal and mirabilite (for Ashi points), anti-swelling and analgesic liquid (2 mL/vial, State Pharmaceutical License No. B20020725), anti-swelling and analgesic patches (4 g/patch, State Pharmaceutical License No. B20020725), all supplied by Yabao Pharmaceutical Group Co. Ltd; Palm Scale (XSZC-01, Kefeng Group Corporation Ltd).

For each acupoint, 0.25 g of the corresponding Chinese medicine fine powder was weighed and mixed with 1 mL of anti-swelling and analgesic liquid to form a paste. The remaining 1 mL of anti-swelling and analgesic liquid was evenly applied to the sand-based medicated pad of the anti-swelling and analgesic patch, and the modulated medicinal paste was placed in the center of the patch.

The application procedure was as follows: after routine local disinfection, the prepared patch was applied to the designated acupoints as follows: for 30 min per session per week, Sanyinjiao (SP6) was focused on, and Shenque (RN8) and Ashi (the two most painful points) were focused on daily, for 6–8 h per session. One treatment course lasted 7 days, and a total of four consecutive courses were administered.

#### 2.6.2 Control group

The placebo anti-swelling and analgesic patches were made of bran powder, flour, and purified water, and the placebo anti-swelling and analgesic liquid was made of pigment (brown) and purified water. Another placebo Chinese medicine alternative was used to replace the original acupoint paste used (see Table 1), following the acupoints, procedure, and treatment times as the treatment group. The placebo patches and liquid were provided by Yabao Pharmaceutical Group Co. Ltd, and the placebo herbal substitute was provided by Shandong Academy of TCM.

**Table 1:** Basic information of placebo herbal substitutes.

Alternatives to traditional Chinese medicine	Main components of alternatives	Similarity test of Chinese medicine with the treatment group	
		Trait similarity (%)	Odor similarity (%)
Sprinkles substitute	Black bean powder, flour, cornmeal, spinach powder, 1/10th dose of finely ground spice in the treatment group	85	80
White mustard substitute	Flour, cocoa powder, corn oil, 1/10th dose of white mustard powder in the treatment group	90	80
*Acorus calamus* substitute	Black bean powder, flour, 1/10th dose of calamus powder in the treatment group	85	80
Chuanxiong substitute	Black bean powder, flour, spinach powder, 1/10th dose of Chuanxiong powder in the treatment group	85	85
Substitute for galangal	Flour, black bean flour, corn flour, spinach flour, pumpkin flour, sunset red pigment, 1/10th dose of galangal powder in the treatment group	80	85
Glauber's salt substitute	Corn starch, 1/10th dose of mannite powder in the treatment group	80	80

### 2.7 Observation indicators

#### 2.7.1 Main outcome indicators

VAS score (11): A 10-point scale was used, with “0” indicating no pain and “10” indicating the most intolerable pain. Patients marked the level that represented the current level of pain. (Points of evaluation: Before and after treatment, assessed two times in total.)

#### 2.7.2 Secondary outcome indicators

TCM syndrome score (12) was used to evaluate the severity of TCM-related symptoms, including hyperpigmentation after herpes subsides, tingling or tampering, disturbed sleep at night, purplish tongue or petechiae, and stringy pulse. Each item is rated as 0 (none), 2 (mild), 4 (moderate), or 6 (severe). The total score reflects overall symptom severity. The higher the score, the more severe the clinical symptoms. (Points of evaluation: before and after treatment, assessed two times in total.)
Pittsburgh Sleep Quality Index (PSQI) (13) was used to evaluate the patient's recent sleep quality across seven items, and the cumulative score of each item is used to calculate the total score of PSQI, with the total score ranging from 0 to 21. The higher the score, the worse the quality of sleep, while a lower score denotes better sleep quality. (Points of evaluation: before and after treatment, assessed two times in total.)
The 36-item Short Form Health Survey (SF-36) (14) score was used to assess the patient's quality of life, including physical health (PCS) and mental health (MCS). Total scores range from 0 to 100, with lower scores indicating worse health status. (Points of evaluation: before and after treatment, assessed two times in total.)
To assess inflammatory factors—monocyte chemotactic protein-1 (MCP-1), interleukin 6 (IL-6), and tumor necrosis factor alpha (TNF-α): 6 mL of fasting elbow venous blood was drawn from patients and centrifuged, and the supernatant was aspirated. Enzyme-linked immunosorbent method (ELISA) was used to test the levels of MCP-1, IL-6, and TNF-α. (Points of evaluation: before and after treatment, assessed two times in total.)
To assess t-cell subpopulations—cluster of differentiation 3 (CD3+), cluster of differentiation 4 (CD4+), and cluster of differentiation 8 (CD8+): 6 mL of fasting elbow venous blood was drawn from patients, and the levels of CD3+, CD4+, and CD8+ were measured using automated flow cytometer. (Points of evaluation: before and after treatment, assessed two times in total.)
To assess neurotransmitters 5-hydroxytryptamine (5-HT), substance P (SP), and β-endorphin (β-EP): 6 mL of fasting elbow venous blood was drawn from patients and centrifuged, and the supernatant was aspirated. ELISA was used to test the levels of 5-HT, SP, and β-EP in blood. (Points of evaluation: before and after treatment, assessed two times in total.)

### 2.8 Criteria for determining efficacy

Referring to the “Guiding Principles for Clinical Research of New Chinese Medicines (for Trial Implementation) (15),” the efficacy evaluation criteria were formulated based on the reduction rate of VAS score. The calculation formula for the VAS score reduction rate is as follows: VAS score reduction rate = (pre-treatment VAS score − post-treatment VAS score)/pre-treatment VAS score × 100%. The criteria for evaluating the therapeutic effect are defined accordingly: a reduction rate of ≥95% indicates a cure; a reduction rate of ≥70% and < 95% represents a significant effect; a reduction rate of ≥30% and <70% means the treatment is effective; and a reduction rate of <30% is deemed ineffective.

### 2.9 Sample size estimation methods and statistical methods

The sample size estimation is conducted using the superiority test formula for a 1:1 allocation between the treatment group and the control group:

$$n=\frac{{\left(z_{a}+z_{R}\right)}^{2}\left[P\underset{C\;\;\;\;\;\;\;\;\;\;\;\;\;\;\;\;\;\;\;\;\;\;\;C}{\left(1-P\right)}+P\underset{T\;\;\;\;\;\;\;\;\;\;\;\;\;\;\;\;\;\;\;\;\;\;\;T}{\left(1-P\right)}\right]}{{\left(P_{T}-P_{C}\right)}^{2}}$$

The preliminary experiment indicated that the efficacy rate of placebo acupoint patch therapy for PHN is 47%. With the application of the anti-swelling and analgesic patch, the expected efficacy rate is 70%. This research adopts a randomized controlled trial (RCT) to compare the efficacy differences between the two treatment methods, with the significance level *α* = 0.05 and the test power 1 − β = 0.8. The final results were obtained using an analyzed sample size of 112 patients. The sample size of the anti-swelling and analgesic acupoint patch treatment group (56 patients) and the placebo acupoint patch control group (56 patients) can achieve 80% test power. Considering the factors of dropout and loss to follow-up, the sample size is expanded by 20%, and the target sample size for each group is determined to be 67, so the total sample size is 134.

### 2.10 Statistical analysis

The primary analysis for between-group comparisons of continuous outcomes was performed using analysis of covariance (ANCOVA). ANCOVA was applied without clear evidence that the assumptions of the model (for instance normality and homogeneity) were tested or verified. Each post-treatment score was modeled as the dependent variable, with the treatment group as a fixed factor and the corresponding baseline score as a covariate. To further control for potential confounding, a pre-specified secondary ANCOVA model was fitted that additionally included the following baseline covariates: age, sex, duration of illness, and study center. This approach ensures that the estimated treatment effect is adjusted for the most clinically relevant prognostic factors. To address potential bias introduced by unequal dropout rates, we performed a sensitivity analysis using multiple imputation. A secondary analysis was conducted to assess the impact of the multicenter design. The homogeneity of the treatment effect across different study centers was explored by including a treatment-by-center interaction term in the ANCOVA model. Results from the primary ANCOVA model (adjusted for baseline only) are presented as the main finding. Results from the sensitivity and multi-covariate models are reported to demonstrate the robustness of the conclusions.

## 3 Results

### 3.1 Comparison of the general information between the two groups of patients

A total of 134 patients with PHN were enrolled, of whom 17 dropped out during the study (10 in the treatment group: 4 due to itching/blistering and 6 due to incomplete treatment; 7 in the control group: 2 due to intolerable pain and 5 due to skin allergy). Ultimately, 57 patients in the treatment group and 60 in the control group completed the study (see Figure 1). Comparison of the two groups of patients’ gender, age, and general information on the course of herpes zoster showed that the difference was not statistically significant (*P* > 0.05), indicating comparability (see Table 2). Baseline characteristics are presented in Table 2. No statistically significant differences were observed between the groups; however, given the relatively small sample size, these results may not fully establish equivalence.

**Table 2:** Comparison of the general information between the two groups of patients.

Groups	Sex (example)		Age (years)			Duration of illness (months)		
	Male	Female	Minimal	Greatest	Average ($\bar{x}\pm s$)	Shortest	Longest	Average ($\bar{x}\pm s$)
Treatment group (*n* = 57)	31	26	19	75	51.19 ± 14.90	3	6	4.61 ± 1.49
Control group (*n* = 60)	27	33	19	75	52.58 ± 13.64	3	6	4.58 ± 1.50

### 3.2 Comparison of VAS scores between the two groups of patients

The baseline VAS scores were comparable (*P* > 0.05). After treatment, the scores of both groups decreased. ANCOVA showed that the treatment group significantly outperformed the control group in reducing VAS scores [Average deviation, which is the average of the absolute values (AMD) = −1.35 points; 95% CI: −1.82 to −0.88; *P* < 0.001]. The sensitivity analysis using multiple imputation for missing VAS data yielded consistent results (AMD = −1.32 points; 95% CI: −1.78 to −0.86; *P* < 0.001) (see Table 3).

**Table 3:** Comparison of VAS scores between the two groups of patients (score, $\bar{x}\pm s$).

Groups	Pre-treatment	Post-treatment	AMD (95% CI)	*P*
Treatment group (*n* = 57)	5.54 ± 1.54	2.35 ± 1.26	−1.35 (−1.82 to −0.88)	<0.001
Control group (*n* = 60)	5.40 ± 1.44	3.67 ± 1.27		

The AMD and *P*-value are derived from ANCOVA, with the baseline TCM syndrome score as the covariate. Negative values indicate that the treatment group had a greater reduction in score.

### 3.3 Comparison of TCM syndrome scores between the two groups of patients

The baseline TCM syndrome scores were comparable (*P* > 0.05). After treatment, the scores of both groups decreased. ANCOVA showed that the treatment group significantly outperformed the control group in reducing TCM syndrome scores (AMD = −3.75 points; 95% CI: −5.18 to −2.32; *P* < 0.001) (see Table 4).

**Table 4:** Comparison of TCM syndrome scores between the two groups of patients (score, $\bar{x}\pm s$).

Groups	Pre-treatment	Post-treatment	AMD (95% CI)	*P*
Treatment group (*n* = 57)	14.70 ± 4.94	6.63 ± 3.87	−3.75 (−5.18 to −2.32)	<0.001
Control group (*n* = 60)	14.47 ± 5.33	10.23 ± 4.47		

The AMD and *P*-value are derived from ANCOVA, with the baseline VAS score as a covariate. Negative values indicate that the treatment group had a greater reduction in score.

### 3.4 Comparison of PSQI scores between the two groups of patients

The baseline PSQI scores were comparable (*P* > 0.05). After treatment, the sleep quality of both groups improved. ANCOVA showed that the treatment group significantly outperformed the control group in reducing the PSQI score (AMD = −1.95 points; 95% CI: −3.18 to −0.72; *P* = 0.002) (see Table 5).

**Table 5:** Comparison of PSQI scores between the two groups of patients (score, $\bar{x}\pm s$).

Groups	Pre-treatment	Post-treatment	AMD (95% CI)	*P*
Treatment group (*n* = 57)	7.88 ± 4.65	4.35 ± 3.27	−1.95 (−3.18 to −0.72)	<0.002
Control group (*n* = 60)	7.83 ± 4.64	6.25 ± 4.18		

The AMD and *P*-value were derived from ANCOVA, with baseline PSQI score as the covariate. Negative values indicate that the sleep quality of the treatment group improved more significantly.

### 3.5 Comparison of SF-36 scores between the two groups of patients

The baseline SF-36 scores were comparable (*P* > 0.05). After treatment, the quality of life scores of both groups increased. ANCOVA showed that the treatment group significantly outperformed the control group in improving the total score of SF-36 (AMD = 6.15 points; 95% CI: 2.01–10.29; *P* = 0.004) (see Table 6).

**Table 6:** Comparison of SF-36 scores between the two groups of patients (score, $\bar{x}\pm s$).

Groups	Pre-treatment	Post-treatment	AMD (95% CI)	*P*
Treatment group (*n* = 57)	108.53 ± 17.77	124.41 ± 16.47	6.15 (2.01–10.29)	0.004
Control group (*n* = 60)	109.33 ± 18.46	118.12 ± 17.12		

The AMD and *P*-value are derived from ANCOVA, with the baseline SF-36 score serving as the covariate. Positive values indicate that the quality of life in the treatment group has improved more significantly.

### 3.6 Comparison of MCP-1, IL-6, and TNF-α levels between the two groups of patients

The baseline levels of inflammatory factors were comparable (*P* > 0.05). After treatment, all the levels of inflammatory factors of both groups decreased. ANCOVA showed that the treatment group was significantly superior to the control group in reducing the levels of MCP-1, IL-6, and TNF-α. (MCP-1: AMD = −2.25 pg/mL, 95% CI: −4.12 to −0.38, *P* = 0.019; IL-6: AMD = −6.58 pg/mL, 95% CI: −8.12 to −5.04, *P* < 0.001; TNF-α: AMD = −3.85 pg/mL, 95% CI: −6.01 to −1.69, *P* = 0.001) (see Table 7).

**Table 7:** Comparison of MCP-1, IL-6, and TNF-α levels between the two groups of patients (pg/mL, $\bar{x}\pm s$).

Groups	MCP-1	IL-6	TNF-α
Treatment group (*n* = 57)			
Pre-treatment	304.88 ± 21.99	47.84 ± 4.74	193.51 ± 7.83
Post-treatment	186.08 ± 13.90	27.49 ± 3.95	108.82 ± 10.47
Control group (*n* = 60)			
Pre-treatment	304.76 ± 22.79	48.07 ± 3.79	194.47 ± 7.50
Post-treatment	188.38 ± 13.62	34.14 ± 4.96	112.72 ± 8.37
AMD (95% CI)	−2.25 (−4.12 to −0.38)	−6.58 (−8.12 to −5.04)	−3.85 (−6.01 to −1.69)
*P*	0.019	<0.001	0.001

The AMD and *P*-values are derived from ANCOVA, with each indicator's baseline value serving as a covariate. For MCP-1, IL-6, and TNF-α, negative values indicate that the treatment group showed a greater decrease.

### 3.7 Comparison of CD3+, CD4+, and CD8+ levels between the two groups of patients

The baseline levels of T-cell subsets were comparable (*P* > 0.05). After treatment, compared with the control group, the treatment group showed significant advantages in increasing the levels of CD3+ and CD4+, and reducing the level of CD8+ (CD3+: AMD = 3.55%, 95% CI: 1.88–5.22, *P* < 0.001; CD4+: AMD = 5.05%, 95% CI: 2.58–7.52, *P* < 0.001; CD8+: AMD = −8.55%, 95% CI: −9.72 to −7.38, *P* < 0.001) (see Table 8).

**Table 8:** Comparison of CD3+, CD4+, and CD8+ levels between the two groups of patients (%, $\bar{x}\pm s$).

Groups	CD3+	CD4+	CD8+
Treatment group (*n* = 57)			
Pre-treatment	32.70 ± 9.47	32.64 ± 8.18	34.15 ± 9.14
Post-treatment	58.21 ± 8.20	42.50 ± 9.10	20.00 ± 4.20
Control group (*n* = 60)			
Pre-treatment	34.67 ± 8.79	34.52 ± 11.50	34.05 ± 12.10
Post-treatment	54.65 ± 5.79	37.38 ± 11.23	28.61 ± 2.01
AMD (95% CI)	3.55 (1.88 to 5.22)	5.05 (2.58 to 7.52)	−8.55 (−9.72 to −7.38)
*P*	<0.001	<0.001	<0.001

The AMD and *P*-values are derived from ANCOVA, with each indicator's baseline value serving as a covariate. For CD3+ and CD4+, positive values indicate that the treatment group showed a greater increase; for CD8+, negative values indicate that the treatment group showed a greater decrease.

### 3.8 Comparison of 5-HT, SP, and β-EP levels between the two groups of patients

The baseline levels of neurotransmitters were comparable (*P* > 0.05). After treatment, compared with the control group, the treatment group showed significant advantages in reducing the level of 5-HT and SP, and increasing the levels of β-EP (5-HT: AMD = −9.98 pg/mL, 95% CI: −11.52 to −8.44, *P* < 0.001; SP: AMD = −4.40 pg/mL, 95% CI: −5.85 to −2.95, *P* < 0.001; β-EP: AMD = 15.48 pg/mL, 95% CI: 11.25–19.71, *P* < 0.001) (see Table 9).

**Table 9:** Comparison of 5-HT, SP, and β-EP levels between the two groups of patients (pg/mL, $\bar{x}\pm s$).

Groups	5-HT	SP	β-EP
Treatment group (*n* = 57)			
Pre-treatment	106.10 ± 6.66	12.99 ± 4.45	34.59 ± 12.29
Post-treatment	85.98 ± 6.68	8.37 ± 3.76	56.86 ± 18.91
Control group (*n* = 60)			
Pre-treatment	104.87 ± 5.20	13.13 ± 4.73	34.87 ± 13.07
Post-treatment	96.01 ± 3.65	12.82 ± 13.96	41.33 ± 13.39
AMD (95% CI)	−9.98 (−11.52 to −8.44)	−4.40 (−5.85 to −2.95)	15.48 (11.25 to 19.71)
*P*	<0.001	<0.001	<0.001

The AMD and *P*-values are derived from ANCOVA, with each indicator's baseline value serving as a covariate. For 5-HT and SP, negative values indicate that the treatment group showed a greater decrease; for β-EP, positive values indicate that the treatment group showed a greater increase.

### 3.9 Comparison of clinical outcomes between the two groups of patients

The total effective rate in the treatment group was 84.21% (48/57), significantly surpassing the control group's rate of 61.67% (37/60) (*χ*^2^ = 10.035, *P* = 0.002) (see Table 10).

**Table 10:** Comparison of effective rates between the two groups of patients (cases, *n*).

Groups	Heal and recover completely	Produce an effect	Validity	Null	Total effective	*χ* ^2^	*P*
Treatment group (*n* = 57)	7 (12.28)	4 (7.02)	37 (64.91)	9 (15.79)	48 (84.21)	10.035	0.002
Control group (*n* = 60)	3 (5.00)	7 (11.67)	27 (45.00)	23 (38.33)	37 (61.67)		

### 3.10 Sensitivity and covariate-adjusted analyses

The results of the primary ANCOVA for the VAS score were robust in the sensitivity analysis using multiple imputation for missing data (AMD = −1.32 points, 95% CI: −1.78 to −0.86, *P* < 0.001). Furthermore, the estimated treatment effect remained virtually unchanged and statistically significant when the model was additionally adjusted for age, sex, duration of illness, and study center (AMD = −1.38 points, 95% CI: −1.91 to −0.85, *P* < 0.001). Formal testing revealed no significant treatment-by-center interaction effect (*P* = 0.22), indicating that the treatment effect was consistent across all participating sites.

### 3.11 Adverse reactions

During the treatment process, itching and blistering occurred in four cases in the observation group and skin allergy in five cases in the control group. As a result, the test was halted. Sterile acupuncture needles were then utilized to puncture the affected areas, extract the fluid, and apply sterile gauze. Participants were instructed to maintain dryness in the local area and avoid rubbing, scratching, and using toiletries or other medications. Subsequently, the skin returned to its normal state after a few days.

## 4 Discussion

The results of this study showed that both the treatment group and the control group acupoint application effectively reduced the degree of pain in patients with PHN of the qi stagnation and blood stasis type, improved TCM symptoms, enhanced sleep quality, improved the quality of life, and reduced the levels of blood MCP-1, IL-6, TNF-α, CD_8+_, 5-HT, SP and elevated the levels of CD_3+_, CD_4+_, and β-EP blood content. The total effective rate was significantly higher in the treatment group than in the control group. The indicators and scores of the patients in the control group did not aggravate or recur, but showed a tendency to improve, indicating that the placebo patches (psychotherapy) were also effective in improving the clinical symptoms of patients with PHN. In terms of safety, although adverse reactions occurred in both groups, the incidence rate was low and no major adverse events occurred after treatment, supporting the fact that the safety of clinical use was good.

PHN is a chronic pathological neuropathic pain that occurs after the disappearance of HZ, but its pathogenesis has not been clarified. Studies have shown that the progression of HZ into PHN is closely related to the increase of IL-6 in blood (16). IL-6 can develop and repair aspects of the nervous system, like nerve growth factor, under normal physiological conditions; however, when the content is too high, it can cause nervous system damage and pain. TNF-α is an initiating factor of inflammatory response, which can also promote Th1 cells to release pro-inflammatory factors such as IL-1β and IL-6, inhibit the release of anti-inflammatory factors such as IL-10, and participate in indirect regulation of macrophage aggregation and neuropathic pain. TNF-α is mainly produced by mononuclear macrophages, glial cells, and nucleus pulposus cells, and it can regulate cell differentiation and growth (17) as well as immune and stress responses. TNF-α levels in patients with PHN are significantly higher than those in normal conditions. It has been demonstrated that (18) PHN is associated with persistently high levels of TNF-α in the blood. In addition, MCP-1, as a chemokine with functions of chemotactic macrophage aggregation and adhesion, can induce central sensitization and participate in the occurrence and maintenance process of inflammatory pain (19). At present, MCP-1 is involved in the occurrence and development of various diseases such as inflammation and tumor. CD3+ refers to total T cells, CD4+ to helper T cells, and CD8+ to suppressor T cells. The number of human T-lymphocyte subsets reflects the individual *T*-cell immune function status (20). When the number and function of T-lymphocyte subsets change, the normal immune system function of the body is abnormal, increasing the probability of virus infection and leading to the occurrence of infectious diseases. 5-HT is an endogenous bioactive substance, distributed in the pineal gland and hypothalamus as a neurotransmitter, and involved in the regulation of physiological functions such as pain, sleep, and body temperature. As an endogenous bioactive substance, it participates in the regulation of pain mechanism in both the peripheral system and central system. Studies have shown that PHN relief is closely related to the inhibition of 5-HT release in the body (21). SP, as a nociceptive neuropeptide released by nerve endings, can participate in pain regulation and transmission of nociceptive information at the spinal cord level, and plays an important role in neuropathological pain process (22). SP in peripheral blood can cause plasma exudation, smooth muscle contraction, vasodilation, and gland secretion; it also stimulates various inflammatory mediators such as prostaglandin, histamine, and kinin release, leading to accumulation of inflammatory and pain-causing substances, and further stimulates afferent fibers of nociceptive information. The pain information is transmitted into the central nervous system to cause pain (23). β-EP is one of the endogenous opioid neurotransmitters that can inhibit the release of SP. It is mainly from the hypothalamus–pituitar*y* axis and is an important part of the body's anti-pain system. When the body is stimulated by pain, endogenous opioid peptide is released (24), inhibiting the pain transmission from primary sensory neurons to spinal cord to relieve pain. Therefore, the improvement of pain degree, TCM syndrome, sleep, and quality of life in patients with PHN is closely related to the increase of blood CD3+, CD4+, and β-EP levels and the decrease of MCP-1, IL-6, TNF-α, CD8+, 5-HT, and SP levels.

TCM believes that PHN is a manifestation of qi deficiency and the presence of pathogenic factors. Long illness depletes qi and yin, causing meridians to lose nourishment. Residual poison persists, pathogenic factors accumulate in the skin, and blood stasis blocks meridians—when the flow of qi and blood is blocked, pain arises. In addition, middle-aged and elderly people are weak in constitution, and long-term pain further depletes qi. Acupoint application is a unique external therapy in TCM. It is based on the theory of TCM and the theory of meridians and acupoints. It selects suitable drugs to be applied to the corresponding acupoints on the patient's body surface. Through meridian conduction, it regulates qi and blood, and restores the balance of yin and yang. TCM enters the human body through the cortex, which not only avoids liver and kidney damage caused by oral western medicine and pain intolerance caused by acupoint injection but is also simple to operate. In addition, acupoint sticking therapy is administered from body surface acupoints or mucous membranes, so abnormal reactions (such as allergy) and tolerance of patients can be observed at any time. Once adverse reactions occur, drugs can be withdrawn immediately to prevent deterioration. Although acupoint application is widely used in clinical practice, there is a lack of high-quality RCTs to confirm its effectiveness and safety. Therefore, this study adopts a multicenter, prospective, randomized, double-blind, placebo-controlled trial to verify the clinical efficacy of acupoint application in the treatment of PHN. The anti-swelling and analgesic patch has been used clinically for many years (25). It consists of external application of anti-swelling and analgesic liquid. Its main components include *Artemisia ormosana* L., menthol, and water vine seed, which have the effect of serving as an anti-swelling and analgesic medicine. Artemisia seeds have the effects of diuresis, dampness absorption, anti-swelling, and analgesic. They can also promote drug penetration, and have good viscosity and water absorption (26). Combined with menthol and water vine in detumescence and pain solution, they can enhance their penetration.

As for the acupoints, Ashi points, Sanyinjiao, and Shen Que were chosen. The Ashi point represents the reaction point of the disease. By applying to this point, the drug acts directly on the site of posterior nerve pain, expelling pathogenic factors and providing rapid pain relief (27). Sanyinjiao belongs to the spleen meridian and is the intersection of the liver, spleen, and kidney meridians. It has the effect of strengthening the spleen and stomach, benefiting the liver and kidney. Research (28) has proved that acupuncture at Sanyinjiao point is effective in treating PHN. The Shenque point, situated at the center of the abdomen, serves as a crucial hub connecting the 8 extraordinary meridians, the 12 regular meridians, and the 6 viscera and 6 bowels. Anatomically, it corresponds to a convergence point of the meridians and the vital energy (qi), encompassing the skin, connective tissue, peritoneal wall, and superficial abdominal wall. Notably, it is primarily associated with the peripheral venous network around the umbilical cord. Unlike other abdominal regions, the Shenque point is characterized by reduced subcutaneous fat but abundant blood supply, close proximity to the peritoneal membrane, and heightened sensitivity, penetration, and absorption rates. This unique anatomical profile renders it an effective site for the application of medicinal patches in the treatment of postherpetic neuralgia (29). These are the commonly used acupoints for drug acupoint placement for the treatment of PHN.

In terms of medicine, six Chinese herbs were selected: white mustard seed, asarone, galangal, mirabilite, calamus, and Chuanxiong. Among them, asarone and white mustard seed constitute one pair, aiding meridian passage and pain relief. Moreover, asarone and white mustard seed can produce a strong stimulating effect on Sanyinjiao in a short time, and can achieve the therapeutic purpose by lasting stimulation of “acupoints, meridians and zangfu organs” (30). Galangal and mirabilite form another pair, as recorded in the Collection of Prescriptions: “Bitter and warm to disperse. Then the liver and kidney are strong and the pain is healed.” The Ashi point is selected based on areas of tenderness, maximizing the local therapeutic effect of Galangal sinken-warm pain relief and mirabilite on clearing tender points, allowing the medicine to reach the affected area directly. Calamus and Chuangxiong form one pair, and the two drugs are combined with occulin, working together to warm, open up, and regulate qi. Moreover, Chuangxiong is an essential drug for treating the pain syndrome of qi stagnation and blood stasis. The rich ligustrazine is absorbed transcutaneously through the Shenque point, which can treat PHN of qi stagnation and blood stasis by regulating the blood flow, resisting platelet aggregation and anticoagulation, and increasing the negative charge of hemoglobin (31). The method has the advantages of strict prescription, direct, special, and rapid effect on disease site, simple operation, safety, and stability, with reduced toxicity. It expels pathogenic factors without harming healthy qi, achieving the therapeutic effects of regulating qi, promoting blood circulation, removing blood stasis, and relieving pain.

The results of this rigorous, multicenter, double-blind, placebo-controlled trial provide compelling evidence that acupoint application is an effective therapy for PHN of the qi stagnation and blood stasis type. Substantively, this study advances the field beyond prior literature by implementing key methodological safeguards: the use of a covariate-adjusted primary analysis (ANCOVA) to control for regression-to-the-mean and baseline imbalances, a double-dummy blinding technique to minimize performance and detection bias, and a multicenter design that enhances the generalizability of our findings. For the increasing number of patients with PHN, the use of acupoint patch therapy offers unique advantages. It can be used in combination with conventional drugs to achieve the clinical effects of reducing patients’ pain, enhancing therapeutic effects, and improving the quality of life. Although ANCOVA was applied to adjust for baseline differences, model assumptions such as normality and homogeneity of variance were not formally tested. Given the sample size, the analysis is considered robust to moderate non-normality; however, this should be verified in future trials.

The multicenter design of this study has expanded the scale of the research sample, making the research results more universal and representative. Furthermore, the application of ANCOVA, adjusted for baseline values, in accordance with current methodological recommendations, strengthens the validity of our between-group comparisons and provides more precise estimates of the treatment effect. However, this study has several methodological limitations that should be acknowledged. First, although between-group differences were statistically significant and analyzed using a robust method (ANCOVA), the clinical meaningfulness of some observed changes should be interpreted with caution. The observed improvements in both groups could partly reflect the natural course of PHN or placebo effects. Second, follow-up was limited to the immediate post-treatment phase, and thus the long-term sustainability of therapeutic effects remains unclear. Third, although we employed ANCOVA to address baseline imbalances and conducted sensitivity analysis for missing data, the dropout rate was relatively high (12.7%) and unbalanced between groups, which remains a potential source of bias. Fourth, the occurrence of skin reaction produced by acupoint application may be related to the frequency of drug application. Continuous application may make the skin unable to restore the original state and lead to skin reactions, which lead to patients dropping out. The frequency of application should be adjusted to reduce the occurrence of adverse reactions. In addition, the VAS scores are inherently subjective, which may introduce a great risk of bias.

Moreover, this study focused exclusively on the qi stagnation and blood stasis subtype of PHN, which is the most common clinical type of PHN. However, PHN can be classified into qi stagnation and blood stasis type PHN, hepatic and heated liver meridians PHN, spleen deficiency and dampness, besides the individual variations among patients. Future research by our team will explore the efficacy of acupoint application in patients with different TCM subtypes. Furthermore, the selection of acupoints and herbal components for acupoint application should be tailored to the specific TCM syndrome type. We will also add simple basic treatment to evaluate the placebo treatment. Further study should also include the evaluation of the efficacy from multi-dimensional directions such as neurology, imaging, and verification of the mechanism of acupoint application in the treatment of this disease by cross-fusion of multiple disciplines in the laboratory, ultimately advancing the development of optimized and syndrome-specific treatment protocols for PHN.

## Data Availability

The raw data supporting the conclusions of this article will be made available by the authors on request without undue reservation.
